# Co-expression of *AaPMT *and *AaTRI *effectively enhances the yields of tropane alkaloids in *Anisodus acutangulus *hairy roots

**DOI:** 10.1186/1472-6750-11-43

**Published:** 2011-04-28

**Authors:** Guoyin Kai, Sheng Yang, Xiuqin Luo, Wentao Zhou, Xueqing Fu, Ang Zhang, Yan Zhang, Jianbo Xiao

**Affiliations:** 1 Laboratory of Plant Biotechnology, College of Life and Environment Sciences, Shanghai, Normal University, Shanghai 200234, China

## Abstract

**Background:**

Tropane alkaloids (TA) including anisodamine, anisodine, hyoscyamine and scopolamine are a group of important anticholinergic drugs with rapidly increasing market demand, so it is significant to improve TA production by biotechnological approaches. Putrescine *N*-methyltransferase (PMT) was considered as the first rate-limiting upstream enzyme while tropinone reductase I (TRI) was an important branch-controlling enzyme involved in TA biosynthesis. However, there is no report on simultaneous introduction of *PMT *and *TRI *genes into any TA-producing plant including *Anisodus acutangulus *(*A. acutangulus*), which is a Solanaceous perennial plant that is endemic to China and is an attractive resource plant for production of TA.

**Results:**

In this study, 21 *AaPMT *and *AaTRI *double gene transformed lines (PT lines), 9 *AaPMT *single gene transformed lines (P lines) and 5 *AaTRI *single gene transformed lines (T lines) were generated. RT-PCR and real-time fluorescence quantitative analysis results revealed that total *AaPMT *(*AaPMT T*) and total *AaTRI *(*AaTRI T*) gene transcripts in transgenic PT, P and T lines showed higher expression levels than native *AaPMT *(*AaPMT E*) and *AaTRI *(*AaTRI E*) gene transcripts. As compared to the control and single gene transformed lines (P or T lines), PT transgenic hairy root lines produced significantly higher levels of TA. The highest yield of TA was detected as 8.104 mg/g dw in line PT18, which was 8.66, 4.04, and 3.11-times higher than those of the control (0.935 mg/g dw), P3 (highest in P lines, 2.004 mg/g dw) and T12 (highest in T lines, 2.604 mg/g dw), respectively. All the tested samples were found to possess strong radical scavenging capacity, which were similar to control.

**Conclusion:**

In the present study, the co-expression of *AaPMT *and *AaTRI *genes in *A. acutangulus *hairy roots significantly improved the yields of TA and showed higher antioxidant activity than control because of higher total TA content, which is the first report on simultaneous introduction of *PMT *and *TRI *genes into TA-producing plant by biotechnological approaches.

## Background

Tropane alkaloids (TA) including anisodamine, anisodine, hyoscyamine and scopolamine are widely used as anticholinergic agents, which act on the parasympathetic nervous system and exclusively exist in *Solanaceous *plants such as *Anisodus, Atropa, Datura, Duboisia, Hyoscyamus*, and *Scopolia *[1-3]. Specially, anisodamine is not as toxic as atropine and has fewer negative effects on the central nervous system than scopolamine [4]. Furthermore, anisodamine and anisodine also showed protective effects on acute lung injury induced by oleic acid and microvascular injury of acute renal failure in rats [5,6].

The hairy root culture system offered many advantages such as high genetic stability, rapid and hormone-free growth, which was considered as a promising way to produce bioactive components from the medicinal plants [7,8]. It has been proven that the application of small scale jar fermenters for culturing hairy roots induced from several *Solanaceous *plants is a very prospective method for production of TA [9-12]. Now TA biosynthetic pathway and key enzyme gene identification have been made clear, so it is possible to enhance TA production in the hairy root culture by biotechnology methods [13-15].

*A. acutangulus *is a Chinese native medicinal plant with higher content of total alkaloids and it has been considered as an attractive plant resource for TA yield [13,16]. But the natural amounts of anisodamine, anisodine and scopolamine are not very high in *A. acutangulus*. However, these alkaloids are all important in phyto-medicine with rapid increasing market demand. Thus, it is essential to improve their yields by biotechnological approaches. [17,18].

**Figure 1:**
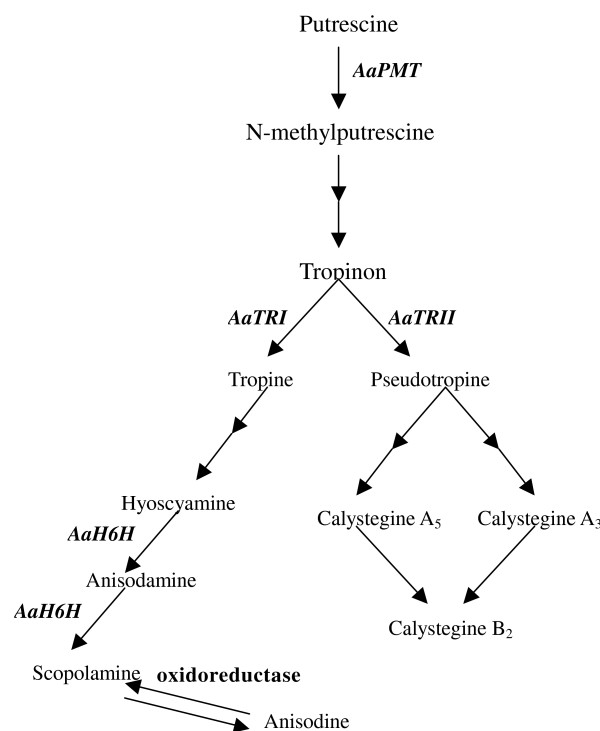
**Schematic biosynthetic pathway of tropane alkaloid in *A. acutangulus***. From the picture, we know that anisodine comes from the change of scopolamine (result is not present).

In TA biosynthetic pathway, Putrescine *N*-methyltransferase (PMT) was considered as the first rate-limiting upstream enzyme while tropinone reductase I (TRI) was an important branch-controlling enzyme (Figure 1). Hence, these two genes are promising target sites for genetic engineering to increase TA in *A. acutangulus *hairy root cultures. Recently, the cDNAs of Putrescine N-methyltransferase (*AaPMT*) and tropinone reductase I (*AaTRI*) of *A. acutangulus *have been successfully cloned by our laboratory [3,19]. However, there is no report on simultaneous introduction of *PMT *and *TRI *into any TA-producing plant including *A. acutangulus*. In this paper, *AaPMT *and *AaTRI *genes were simultaneously introduced into *A. acutangulus *hairy roots for the first time.

## Results

### Identification of transformed root lines through PCR analysis

**Figure 2:**
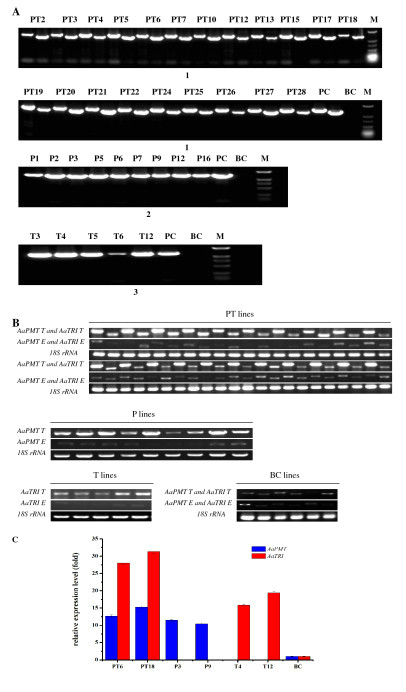
**DNA identification and gene transcripts analysis**. A) PCR analyses of transgenic hairy root lines. Analyses for the presence of *AaPMT *(1269 bp) *and AaTRI *genes (1074 bp) in PT lines (1), *AaPMT *gene in P lines (2) and *AaTRI *gene in T lines (3), respectively. M, DL-2000 Marker. PC, positive control (vector contains corresponding gene). BC, blank control (hairy root generate from blank-vector transformation). B) Effects of transformation on the expression of related genes. *AaPMT T *and *AaTRI T *represent total gene of *AaPMT *and *AaTRI *respectively, *AaPMT E *and *AaTRI E *represent endogenous *AaPMT *and *AaTRI *respectively. C) Real-time fluorescence quantitative PCR analysis of the expressions of *AaPMT *and *AaTRI *in transgenic hairy roots.

Three plasmids containing the cDNAs encoding *AaPMT *and/or *AaTRI*, driven by CaMV 35S promoter, were separately introduced into *A. acutangulus *using disarmed *A. tumefaciens *C58C1 strain. In total, 25 *AaPMT *single gene transformed lines (P lines), 19 *AaTRI *single gene transformed lines (T lines) and 46 *AaPMT *and *AaTRI *double gene transformed lines (PT lines) were generated, respectively (Table 1). The hairy root lines were subcultured for about 4 weeks in hormone-free, B5 solid medium with cefotaxime. At last, 17 P, 15 T and 36 PT hygromycin-resistant hairy root lines were obtained (Table 1). DNA of all the independent hairy roots was isolated and used for PCR analysis using primers specially designed to overlap part of the *AaPMT *or *AaTRI *and the CaMV 35S promoter sequences. The plasmid 1304^+^-*AaPMT *or 1304^+^-*AaTRI *was used as positive control (PC) and control hairy-root lines generated from blank-vector transformations were used as BC. In total, the PCR-positive clones amounted to 52.94% (9/17), 33.33% (5/15) and 58.33% (21/36) for P, T and PT lines, respectively. None of the checked DNA bands was detected from BC lines (Figure 2A).

**Table 1:** Positive clones of transgenic hairy roots.

	Number of root lines			
**Gene constructs**	**Total**	**Hygromycin-resistant**	**PCR-positive**	**Established root lines**

*AaPMT*	25	17	9	P1 P2 P3 P5 P6 P7 P9 P12 P16

*AaTRI*	19	15	5	T3 T4 T5 T6 T12

*AaPMT-AaTRI*	46	36	21	PT2 PT3 PT4 PT5 PT6 PT7 PT10 PT12 PT13 PT15 PT17 PT18 PT19 PT20 PT21 PT22 PT24 PT25 PT26 PT27 PT28

### Morphological characterization of the transgenic hairy roots

**Figure 3:**
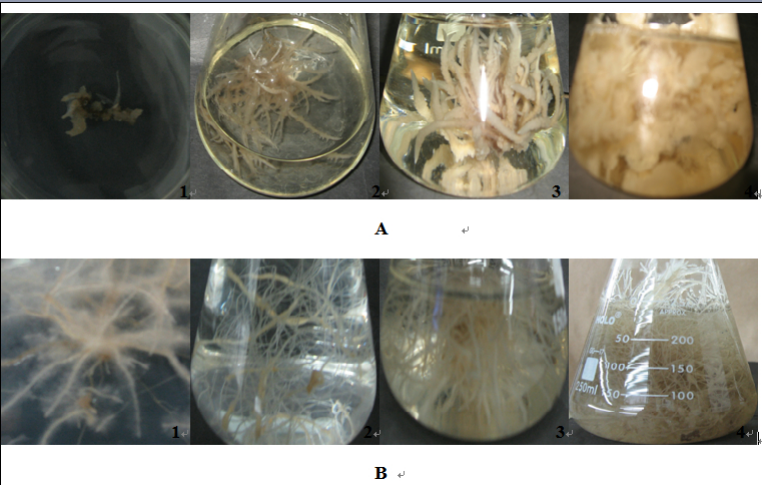
**The morphology of transgenic *A. acutangulus *hairy roots**. A) Abnormal clones. B) Normal clones.

In this experiment, the transgenic hairy roots had two main morphology forms. One form was abnormal and looked brownish, thick with short branches such as PT3, T3 and T5. This kind of hairy root has been abnormal with slow growth since it was cultured on solid medium during the stage of sterilization (Figure 3A 1-4). Contrarily, most of transgenic hairy roots belonged to another form, which were normal and looked yellowish or light brownish with whiter slender branched. These hairy root clones have been always normal from the stage of sterilization in the culture dishes to the stage of rapid growth in the flasks (Figure 3B 1-4).

### Analysis of the transcript level of *AaPMT *and *AaTRI*

Total RNA was isolated from the positive root lines and RT-PCR, real-time fluorescence quantitative analysis were used to determine the expression levels of *AaPMT *and *AaTRI*. As shown in Figure 2B and 2C, the different expression levels illustrated that total *AaPMT *gene (*AaPMT T*) and total *AaTRI *gene (*AaTRI T*) transcripts in transgenic PT, P lines and T lines exhibited higher expression than the native *AaPMT *gene (*AaPMT E*) and native *AaTRI *gene (*AaTRI E*) transcripts. Moreover, the expression patterns of *AaPMT E *and *AaTRI E *in the above transgenic lines showed similar levels with *AaPMT T *and *AaTRI T *in BC lines. On the contrary, the *AaPMT T *and *AaTRI T *transcripts in the P, T and PT lines had higher transcript levels, which demonstrated that all the expression cassettes of the cDNAs encoding *AaPMT *and/or *AaTRI *were introduced into *A. acutangulus *and expressed at varying degrees in corresponding transgenic lines.

Two independent transgenic lines with *AaPMT *and *AaTRI *genes, two lines with *AaPMT *gene and two lines with *AaTRI *gene were chosen (Figure 2C). The average expression levels of *AaPMT *and *AaTRI *gene in PT6 and PT18 were 13.95-fold and 29.63-fold higher when compared with control, respectively. In P3 and P9, the mean expression level of *AaPMT *gene was 10.93-fold higher as compared to control. In T4 and T12, the expression levels of *AaTRI *gene on average were 17.59-fold higher when compared with non-transgenic clones.

### TA profile in transgenic hairy roots

**Figure 4:**
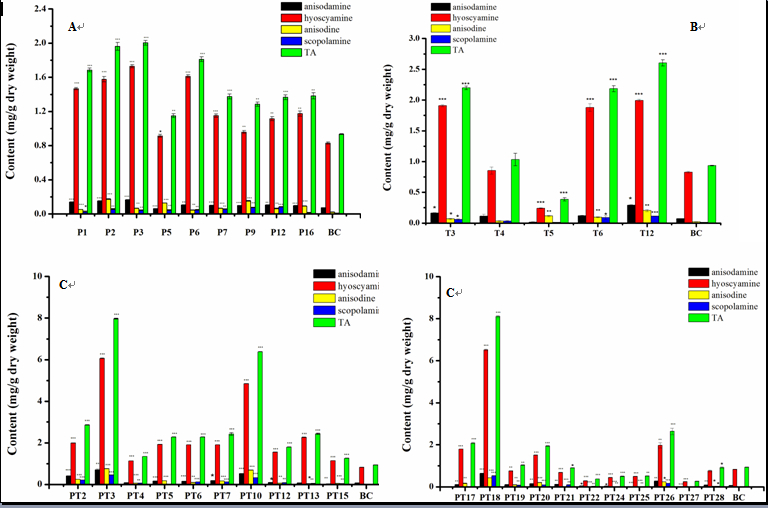
**TA production analyzed by HPLC**. A) TA content in transgenic P lines. B) TA content in transgenic T lines. C) TA content in transgenic PT lines. BC, control hairy root cultures generate from blank-vector transformation. The values are means ± S.D of triplicate analyses. *, **, and *** Significant difference at P < 0.05, 0.01, and 0.001 respectively.

**Figure 5:**
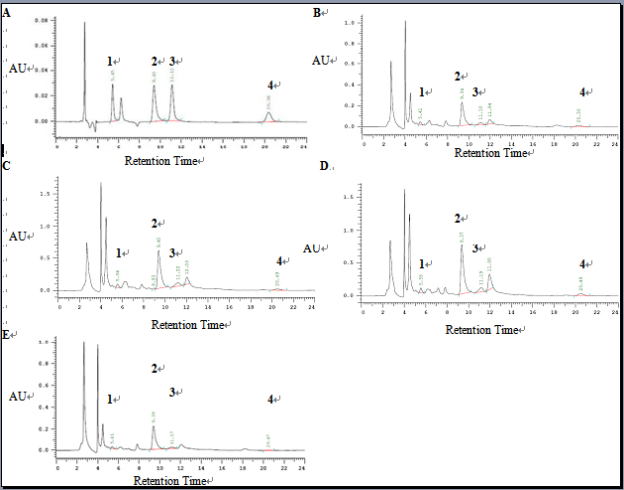
**HPLC chromatograms of TA**. A) mixture of standards of anisodamine, hyoscyamine, anisodine and scopolamine. B) HPLC chromatograms of TA in line P3. C) HPLC chromatograms of TA in line T12. D) HPLC chromatograms of TA in line PT18. E) HPLC chromatograms of TA in line BC1 (TA peaks, 1 for anisodamine, 2 for hyoscyamine, 3 for anisodine and 4 for scopolamine).

The contents of TA in *A. acutangulus *hairy root lines were analyzed by HPLC. Three independent samples extracted from the transgenic lines and the control lines were tested with SPSS software. One sample *t *test was used to identify the accumulations of TA which showed significant changes in different lines and control. The capacities of transgenic root lines to yield TA were shown in Figure 4. P lines showed higher level of TA content than control (0.935 mg/g dw), which ranged from 1.151 to 2.004 mg/g dw (Figure 4A, 5B and 5E). T lines also produced larger level of TA (1.682 mg/g dw) than the control (Figure 5C and 5E, Table 2). Specially, the yield of TA in line T12 reached to 2.604 mg/g dw (Figure 4B). The average content of TA in PT lines was significantly enhanced, which was much higher than the average content of the control. The levels of TA in PT lines were within the range from 0.263 to 8.104 mg/g dw. The highest yield of TA was detected as 8.104 mg/g dw in line PT18, which was 8.66, 4.04, and 3.11-times higher than those of the control (0.935 mg/g dw), P3 (highest in P lines, 2.004 mg/g dw) and T12 (highest in T lines, 2.604 mg/g dw) (Figure 4C, 5D and 5E). The accumulations of scopolamine and anisodine were obviously improved in PT18 (P < 0.001). The yields of scopolamine and anisodine in PT18 were 49-folds and 19- folds higher than the amounts in the BC line, respectively. Moreover, the content of single alkaloid among all hairy root lines was in accordance with the content of TA (Figure 4A, B and 4C). For example, the content of TA in line PT18 was significantly higher compared with that in the control lines (P < 0.001). So the contents of four alkaloids in line PT18 were also much higher than control. The result above confirmed that biotechnological approach was a high-efficiency way to enhance the production of tropane alkaloids.

**Table 2:** The average productions of transgenic lines.

	*Anisodamine*	*Hyoscyamine*	*Anisodine*	*Scopolamine*	Total TA
P lines	0.114 ± 0.001	1.300 ± 0.006	0.094 ± 0.002	0.0503 ± 0.001	1.558 ± 0.036
T lines	0.141 ± 0.010	1.376 ± 0.03	0.104 ± 0.004	0.0611 ± 0.011	1.682 ± 0.050
PT lines	0.193 ± 0.01	1.914 ± 0.021	0.167 ± 0.005	0.120 ± 0.003	2.395 ± 0.016

BC lines	0.0738 ± 0.0005	0.829 ± 0.047	0.0218 ± 0.0003	0.0106 ± 0.0005	0.935 ± 0.051

The average productions of anisodamine, hyoscyamine, anisodine, scopolamine and total TA among various transgenic hairy root lines (content unit: mg/g dry weight)

The difference of average yields of TA in P, T, PT, and BC lines was showed in Table 2. It illustrated that the content of TA in PT lines (2.395 mg/g dw) was the highest, when compared with T lines (1.682 mg/g dw), P lines (1.558 mg/g dw) and BC lines (0.935 mg/g dw). The average content of hyoscyamine in PT lines (1.914 mg/g dw) was much higher than that of BC lines (0.829 mg/g dw), but there is no remarkable difference between T (1.376 mg/g dw) and P lines (1.300 mg/g dw). The average content of anisodine in PT lines (0.167 mg/g dw) showed further higher than that of BC lines (0.0218 mg/g dw). The average content of anisodine was almost the same between T (0.104 mg/g dw) and P lines (0.0937 mg/g dw). To scopolamine, its average content in PT lines was 0.12 mg/g dw, which also showed much higher than the content in BC lines (0.0106 mg/g dw). It was improved by inconceivable 11.32 times compared to the BC lines.

### Antioxidant activity analysis of transgenic hairy roots

**Figure 6:**
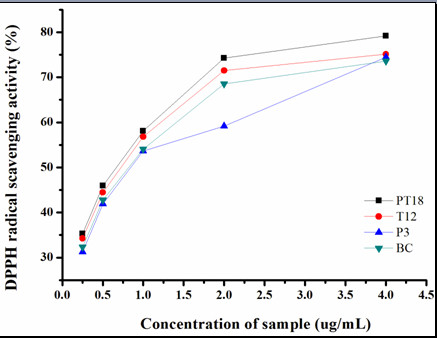
**DPPH scavenging activities of methanolic TA from four different hairy root lines (P3, T12, PT9 and BC)**. Results represent means ± standard deviation (n = 3).

The antioxidant activity of TA from hairy roots was estimated by measuring DPPH radical scavenging in this study. As shown in Figure 6, all the tested samples were found to possess strong radical scavenging capacity, which were similar to control.

## Discussion

TA production in *A. acutangulus *hairy root cultures was indeed enhanced by biotechnology approach. However, in present study, considerable variation in morphology between different lines was observed, which was similar to the result reported by Jouhikainen et al [20]. The change of external conditions such as the nuances of ingredient in different 1/2 MS liquid nutrient media, the nuance of temperature in the gyratory shaker and the diversification of internal conditions may lead to the different root morphologies. [21]. However, in our experiment, the yields of TA in abnormal hairy roots were higher than those of normal ones. But they would be broken down when inoculated in liquid medium for some time and they were not routinely subcultured. The results implied that root morphology could lead to the content change of secondary metabolite production [1].

The expression levels of the *AaPMT T *varied from line to line but correlated with their corresponding content of TA in P lines. The average content of TA in P lines showed higher than that of BC lines. Moreover, the contents of anisodamine, anisodine, hyoscyamine and scopolamine were all improved in contrast to those in BC lines, respectively. These results showed that *AaPMT *gene in P lines has a positive influence on the flow of metabolites through TA biosynthesis pathway in *A. acutangulus*, which suggested that *AaPMT *gene is a new promising target site for metabolic regulation of tropane-alkaloid pathway in *A. acutangulus *[21]. However, overexpression of *PMT *gene in *A. belladonna *and *H. niger *hairy root cultures have no significant promotion on accumulation of TA [1,22], which may be due to species-related or different specific post-translational regulation of the native enzyme in respect to the foreign one [23]. Sometimes, genetic manipulation of single enzymes to increase flux through the pathway may display various and even paradox results between different species [24].

The yield of TA in T lines was higher than the control and even P lines, which accounted for that *AaTRI *gene is a more effective regulatory target than *AaPMT *gene for improving metabolic flux in TA biosynthetic pathway. The average content of TA in T lines (1.682 mg/g dw) were higher than that in P lines (1.558 mg/g dw) or BC lines (0.935 mg/g dw). So strong expression of *AaTRI *T in *A. acutangulus *hairy roots could up-regulate content of TA [19]. As shown in Figure 1, two tropinone reductases (TRI and TRII) formed the branching point of tropane alkaloid biosynthesis. High expression of *TRII *gene would lead to increasing accumulation of calystegines in the roots; while strong expression of *TRI *gene was accompanied with increased level of TA and decreased level of calystegine [25]. In a word, high TRI activity in T lines has positive effect on the conversion from pseudotropine to tropine, which would provide more key precursor for synthesis of TA.

Overexpression of single gene encoding a key enzyme may increase flux through the pathway, but its effect may be limited by other rate-committee step to some degree. Therefore, regulation of two or multiple genes would be more suitable to achieve significant gains in product accumulation. This may especially be true in branched pathways in which precursors can be channeled into a variety of metabolites away from the desired product [24]. In our work, line PT18 was the most successful one for production of TA, which was attributed to co-overexpression of two key genes (*AaPMT *and *AaTRI*). These results showed *AaPMT *and *AaTRI *make cooperative effect than single gene in the accumulation of TA in PT lines. In the hairy root lines, overexpressing upstream key enzyme and downstream branch-controlling enzyme may act as a push-pull effect in which flux is pushed toward the branch point by *AaPMT *and then pulled toward the desired product by *AaTRI *(Figure 1).

The DPPH (1,1-Diphenyl-2-picrylhydrazyl) radical scavenging activity, based on the reduction of the stable radical DPPH to yellow-colored diphenylpicrylhydrazine in the presence of a hydrogen donor, was widely used to evaluate the antioxidant activity due to its advantage of rapid and simple measure [26]. TA has shown to possess the antioxidant activities [27]. The antioxidant activity of TA from hairy roots was estimated by measuring DPPH radical scavenging in this study. All the tested samples were found to possess strong radical scavenging capacity, which suggested that there were no significant differences among them. It indicated that antioxidant activity of aliquots of TA remained stable, so transgenic hairy root lines P3, T12 and PT18 showed higher total antioxidant activity of TA due to higher total TA content they produced.

Hyoscyamine-6-hydroxylase (H6H) catalyzes the oxidative reactions in the biosynthetic pathway leading from hyoscyamine to scopolamine and is also an effective regulatory site for the synthesis and accumulation of TA [1,20]. Therefore, in order to enhance the levels of TA and the capacity to convert hyoscyamine to much more scopolamine and anisodine, co-overexpression of multiple biosynthetic genes such as *AaPMT*, *AaTRI *and *AaH6H *in *A. acutangulus *may be a new promising strategy in the near future. In addition, anisodine is also an imperative tropane alkaloid in the market, which was first produced in hairy root cultures. This result suggested anisodine could be biosynthesized by TA biosynthesis pathway, which provided helpful information for our ensuing research.

## Conclusion

In the present study, co-overexpression of *AaPMT *and *AaTRI *genes in *A. acutangulus *hairy roots led to significantly increased production of four kinds of TA and showed higher antioxidant activity than control because of higher total TA content. This is the first report on simultaneous introduction of *PMT *and *TRI *genes into TA-producing plant using biotechnological methods. Our study results showed that transgenic hairy root culture system is a promising approach for improvement and large-scale production of TA in the future.

## Methods

### Construction of Plant Expression Vectors

**Figure 7:**
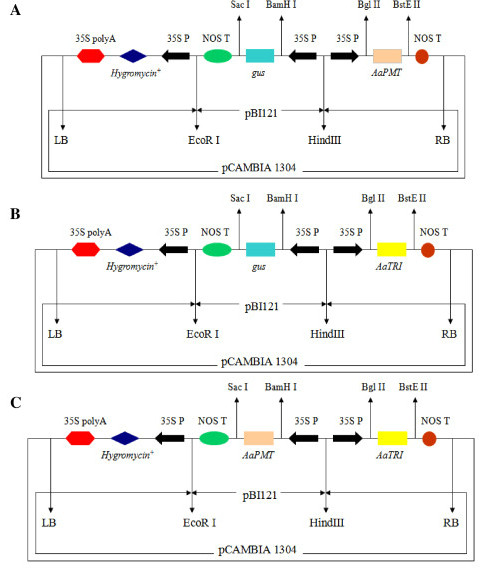
**The construction of recombinant vectors (Kai et al. 2010).** A) pCAMBIA1304^+^-*AaPMT*. B) pCAMBIA1304^+^-*AaTRI*. C) pCAMBIA1304^+^-*AaPMT *-*AaTRI*. The *AaPMT *and *AaTRI *cDNAs are driven by the Cauliflower mosaic virus promoter (35S). The direction of transcription is marked with arrowheads. The restriction enzyme sites *BamH I*, *Sac I*, *Bgl II *and *BstE II *were signed.

The vectors pBI121 (Clontech) and pCAMBIA1304 (CAMBIA) were double-digested with *HindIII *and *EcoRI*. The purified smaller DNA fragment containing a GUS expression cassette from pBI121 was cloned into the large pCAMBIA1304 fragment to generate the recombinant plasmid pCAMBIA1304^+ ^[19]. The full-length *AaPMT *cDNA was inserted into pCAMBIA1304^+ ^in place of the *mGFP5 *and *GUSA *genes to generate pCAMBIA1304^+^-*AaPMT *expression vector containing *AaPMT *gene under the digestion of *BgLII *and *BstEII *(Takara Biotech Co., Ltd) (Figure 7A). Similarly, the pCAMBIA1304^+^-*AaTRI *was also constructed in place of the *mGFP5 *and *GUSA *genes under the digestion of *BgLII *and *BstEII *(Figure 7B). On the basis of pCAMBIA1304^+^-*AaTRI*, the full-length *AaPMT *cDNA was used to replace GUS gene in pCAMBIA1304^+^-*AaTRI *under the digestion of *SacI *and *BamHI *(Takara Biotech Co., Ltd) to generate the expression vector pCAMBIA1304^+^-*AaPMT*-*AaTRI *containing both *AaPMT *and *AaTRI *genes (Figure 7C). The genes *AaPMT *or/and *AaTRI *were under the control of strong cauliflower mosaic virus (CaMV) 35S promoter. The blank vector pCAMBIA1304^+ ^without exogenous gene (such as *AaPMT *or *AaTRI*) was used as the control.

### Plant Transformation and Root Cultivation

**Figure 8:**
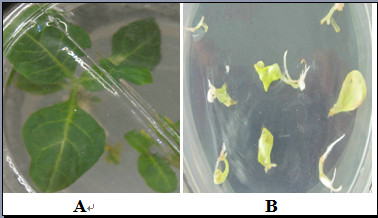
**The explant used for hairy root induction**.

The disarmed A. tumefaciens strain C58C1 harbouring both the A. rhizogenes Ri plasmid pRiA4 [19] and each of the four plasmids constructed above, were used for plant genetic transformation. Different explants including leaf blades, petioles or stems were isolated from 4-week-old *in vitro *grown sterile seedlings of *A. acutangulus *(Figure 8). The isolated explants were cut into small pieces (about 1 cm, 20 pieces per dish) followed by pre-incubation on hormone-free MS containing 8.0 g/L agar plates in the dark for 2 days, and then inoculated with C58C1 bacterial strain for 15 minutes. The explants were dried by blotting with sterile filter paper and then placed back on their original culture plates for cocultivation in the dark for 2 days. Then, the explants were rinsed with sterile water, and transferred onto B5 medium supplemented with 500 mg/L cefotaxime sodium to eliminate the residual *Agrobacterium*, 0.5 mg/L hygromycin to select positive hairy roots. The apical tips of hairy root induced from transformed explants were excised and sub-cultured on B5 solid medium with 500 mg/L cefotaxime at 2-week intervals, the concentration of cefotaxime was gradually lowered and finally omitted after 3 months. When cultures had been cleared of Agrobacterium, hairy roots were transferred onto hormone-free B5 solid medium without cefotaxime [19,28].

### DNA extraction and PCR analysis

When the engineered hairy roots grew to about 5 cm, genomic DNA was isolated from hairy root samples by using DNA pure Plant Kit (Tiangen Biotech Co., Ltd, Beijing, China), which was used in PCR analysis for detecting the presence of *AaPMT *and/or *AaTRI *in transgenic hairy root cultures (Table 3).

**Table 3:** Primer Pairs Employed for the PCR Amplification of TA Biosynthetic Genes *AaPMT *and *AaTRI *

*Primer Pairs 35S PROM 252F*(*Forward*)*and KR of *AaPMT *and *AaTRI(*Reverse*)	
*AaPMT*	Forward: 5'-ATGGAGGTCATAAGCAACCAC-3' Reverse: 5'-TCAAAATTCAACCAAATCCC-3'

*AaTRI*	Forward: 5'-ATGGGAGAATCAAAAGTTTACAT-3' Reverse: 5'-TCAAAATCCACCATTAGCTGTGA-3'

### RNA extraction and gene expression analysis by RT-PCR and real-time fluorescence quantitative analysis

The transgenic hairy roots identified by PCR analysis were chosen and inoculated into 150 mL 1/2MS liquid nutrient media (pH 5.8) in 250 mL conical flasks on a gyratory shaker operating at 100 rpm at 27°C in darkness for RT-PCR and real-time fluorescence quantitative after 60 days' culture. RNA was extracted using RNA pure Plant Kit (Tiangen Biotech Co., Ltd, Beijing, China) following the manufacture's instructions. RNA was quantified using spectrophotometric measurements and kept at -80°C for further analysis [29]. Aliquots of total RNA (1 μg) was used as a template to generate cDNA using avian myeloblastosis virus (AMV) reserve transcriptase (Promega, USA) and then for further semi-quantitative RT-PCR to quantify gene expression profiles of different samples. RT-PCR was carried out using similar procedure as reported before [3,19]. *AaPMT*KF and *AaPMT*KR, *AaTRI*KF and *AaTRI*KR (Table 4) were used to detect the expression of total (including native and introduced cDNA) *AaPMT *and *AaTRI *genes, whereas *AaPMT*KF and *AaPMT *3'-UTR; *AaTRI*KF and *AaTRI *3'-UTR (Table 5) were only used for the expression profiles of native *AaPMT *and *AaTRI *genes, respectively. Meanwhile, RT-PCR with primers 18SF (5'-CCAGGTCCAGACATAGTAAG-3') and 18SR (5'-GTACAAAGGGCAGGGACGTA-3'), designed on the basis of the conserved regions of plant house-keeping genes (18S rRNA genes), was also performed to normalize equal amounts of RNA among samples as an internal standard [30,31]. Gene-specific primers were designed using primer Express 3 (Applied Biosystem Co., Ltd, USA) for real-time fluorescence quantitative: *AaPMT*26F (5'-TGGCAGCACCACCAAAATTA-3') and *AaPMT*163R (5'**-**TGGCCAGAGTGCGCTAAACT**-**3'**) **to detect the expression level of *AaPMT*, *AaTRI*515F (5'-TGCTTCCAAAGCTGCAATAA-3') and *AaTRI*595R (5'-TAAAATGATTCCCGGAGCAA-3') to detect the expression level of *AaTRI*, and 18SF' (5'-GCCTTCGGGATCGGAGTAAT-3') and 18SR' (5'-CCCCCAACTTTCGTTCTTGA-3') to detect the expression level of *18S *(plant house-keeping gene). Real-time RT-PCR analysis was performed in an Applied Biosystem StepOne (Applied Biosystem Co., Ltd, USA). The relative Ct (threshold cycle value) method was used to estimate the initial amount of template present in the reactions by Applied Biosystems SDS 2.0.

**Table 4:** Primer Pairs Employed for the RT-PCR Amplification of Total Biosynthetic Genes *AaPMT *and *AaTRI *

*Primer Pairs: KF of *AaPMT, AaTRI(*Forward*) *and KR of *AaPMT, AaTRI(*Reverse*)	
*AaPMT*	Forward: 5'-ATGGAGGTCATAAGCAACCAC-3' Reverse: 5'-TCAAAATTCAACCAAATCCC-3'

*AaTRI*	Forward: 5'-ATGGGAGAATCAAAAGTTTACAT-3' Reverse: 5'-TCAAAATCCACCATTAGCTGTGA-3'

**Table 5:** Primer Pairs Employed for the RT-PCR Amplification of Native Biosynthetic Genes *AaPMT *and *AaTRI *

*Primer Pairs KF of *AaPMT, AaTRI (*Forward*) *and 3'UTR of *AaPMT, AaTRI	
*AaPMT*	Forward: 5'-ATGGAGGTCATAAGCAACCAC-3' 3-UTR 156R: 5'-ATATCAGTTTATTGCATTATAC-3'

*AaTRI*	Forward: 5'-ATGGGAGAATCAAAAGTTTACAT-3' 3-UTR 244R:5'-TTGCGACATTTTATTGTGATGA-3'

### TA production in transgenic *A. acutangulus *hairy roots by HPLC analysis

The extraction of TA was conducted using the method reported previous [26]. After extraction, HPLC analysis was performed on a HITACHI L2000 HPLC system equipped with a photodiode array detector. The separation of TA was carried out on a reversed-phase symmetry column (250 mm × 4.6 mm; 5 μm). The mobile phase consisted of 22% acetonitrile (HPLC grade) and 78% diethylamin buffer (containing 0.7% diethylamine and adjust pH to 7.2 with orthophosphoric acid). The flow rate was 1.0 mL/min and the injection volume was 20 μL. Four TA compounds including anisodamine, hyoscyamine, anisodine and scopolamine were detected and quantified by comparison with authentic standard curves and retention times.

### Measurement of DPPH free-radical scavenging activity

The free radical scavenging activity of TA from transgenic hairy roots (P3, T12, PT18 and BC) was measured by the DPPH method reported by Adeolu et al. (2008) with slight modifications [32]. Briefly, the different concentration (0.25-4 μg/mL) of 1 mL of each sample was added to 1 mL DPPH (0.2 mM) solution in methanol. The tubes with reaction mixture were vortexed thoroughly and incubated at room temperature for 30 min. Spectrophotometer was used to detect the absorbance of the mixture at 517 nm. The DPPH solution in methanol (0.2 mM) was used as the control. Radical scavenging activity of TA was calculated according to the following formula: DPPH radical scavenging activity (%) = [1 - absorbance of the sample/absorbance of the control] ×100.

### Statistical analysis

All the experiments including PCR identification, semi-quantitative RT-PCR, HPLC and measurement of antioxidant activity were repeated three times. Analysis was repeated three times. Results of TA content are presented as mean values ± S.D. The statistical significance of TA difference was analyzed by one sample *t *test using SPSS software (SPSS, Inc.).

## Authors' contributions

GK designed the study, performed the experiments and revise the manuscript. SY performed the experiments, analyzed the data and draft the manuscript. XL, WZ, XF, AZ and YZ helped to culture hairy roots. JX helped to direct data analysis. All authors read and approved the final manuscript.
